# The genome sequence of the common grass-veneer, *Agriphila tristella *(Denis & Schiffermüller, 1775)

**DOI:** 10.12688/wellcomeopenres.18568.1

**Published:** 2022-12-16

**Authors:** Douglas Boyes, University of Oxford and Wytham Woods Genome Acquisition Lab, Darwin Tree of Life Barcoding collective, Wellcome Sanger Institute Tree of Life programme, Wellcome Sanger Institute Scientific Operations: DNA Pipelines collective, Tree of Life Core Informatics collective, Louis Parkerson, Darwin Tree of Life Consortium

**Affiliations:** 1 UK Centre for Ecology and Hydrology, Wallingford, Oxfordshire, UK; 2 Independent researcher, Norwich, Norfolk, UK

**Keywords:** Agriphila tristella, common grass-veneer, genome sequence, chromosomal, Lepidoptera

## Abstract

We present a genome assembly from an individual male *Agriphila tristella* (the common grass-veneer; Arthropoda; Insecta; Lepidoptera; Crambidae). The genome sequence is 802 megabases in span. Most of the assembly (99.83%) is scaffolded into 23 chromosomal pseudomolecules with the Z sex chromosome assembled. The mitochondrial genome was also assembled and is 15.3 kilobases in length.

## Species taxonomy

Eukaryota; Metazoa; Ecdysozoa; Arthropoda; Hexapoda; Insecta; Pterygota; Neoptera; Endopterygota; Lepidoptera; Glossata; Ditrysia; Pyraloidea; Crambidae; Crambinae; *Agriphila; Agriphila tristella* (Denis and Schiffermüller, 1775) (NCBI: txid1594226).

## Background

The common grass-veneer *Agriphila tristella* (Denis & Schiffermüller, 1775) is a micro-moth of the Crambinae subfamily. It can usually be recognised by its yellow median streak on the forewing which branches into four ‘fingers’ towards the apex of the wing. However, the species can be quite variable and difficult to separate from *Agriphila selasella*. In these cases, the prominent facial cone and differences in the genitalia can be used to identify *A. tristella* reliably ( Lewis, 2012). The species is common in grassland and rough meadows throughout the British Isles, where the eggs are laid on various grasses.

*A. tristella* larvae can be found from September to June, feeding in a vertical silken gallery along the lower part of a grass stem. Pupae can then be found in June and July within oval frass-covered silken cocoons in loose soil amongst the grass roots. The adults typically fly between late June to mid-September, with a peak in August. During this time, they can be readily disturbed by day or attracted to light at night ( Langmaid *et al.,* 2018).

The genome of the common grass-veneer was sequenced as part of the Darwin Tree of Life Project, a collaborative effort to sequence all the named eukaryotic species in the Atlantic Archipelago of Britain and Ireland.

## Genome sequence report

**Figure 1.**
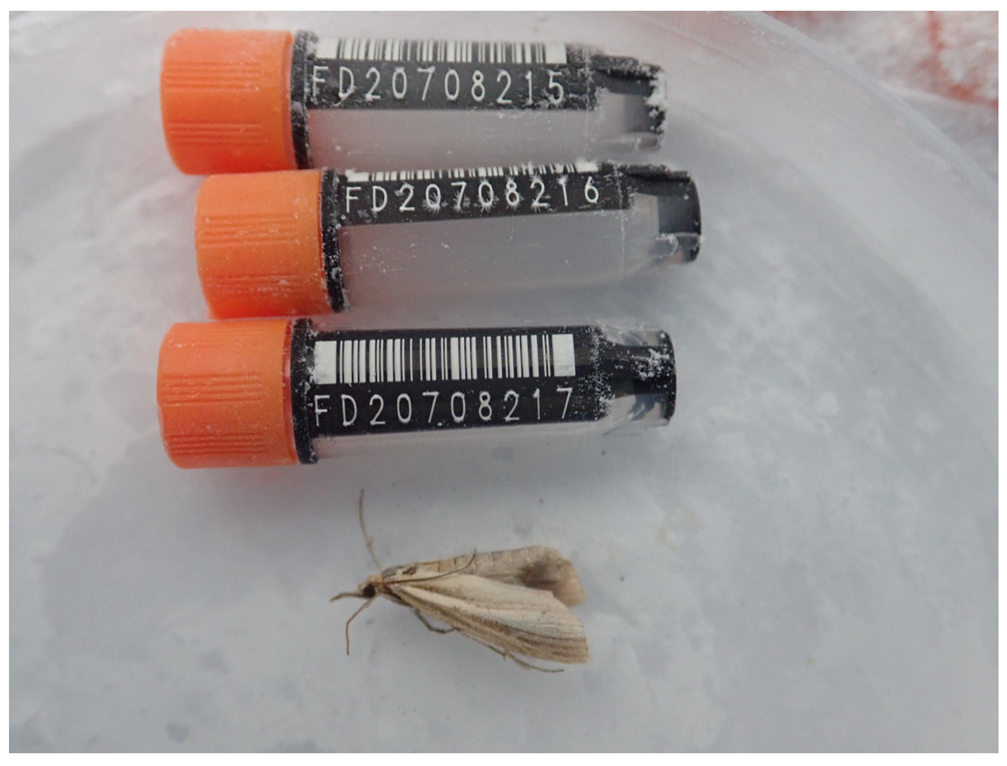
Image of the *A. tristella* specimen taken prior to preservation and processing.

The genome was sequenced from a single male *A. tristella* ( Figure 1), collected in Wytham Woods, Oxford, Berkshire, UK. A total of 29-fold coverage in Pacific Biosciences single-molecule HiFi long reads and 56-fold coverage in 10X Genomics read clouds were generated. Primary assembly contigs were scaffolded with chromosome conformation Hi-C data. Manual assembly curation corrected 86 missing/misjoins and removed 40 haplotypic duplications, reducing the assembly size by 3.91% and the scaffold number by 39.22%, and increasing the scaffold N50 by 32.77%.

**Figure 2.**
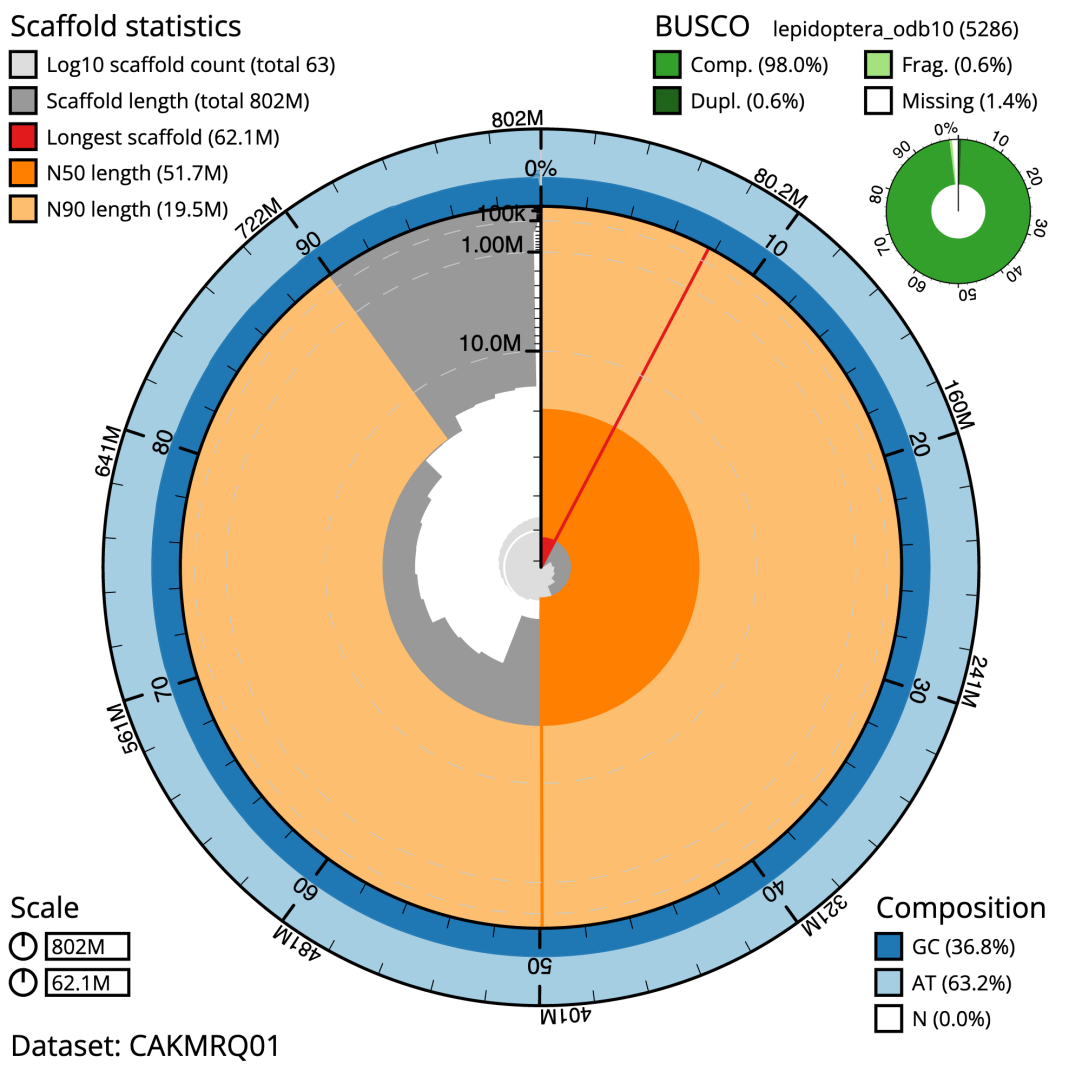
Genome assembly of *A. tristella*, ilAgrTris1.1: metrics. The BlobToolKit Snailplot shows N50 metrics and BUSCO gene completeness. The main plot is divided into 1,000 size-ordered bins around the circumference with each bin representing 0.1% of the 801,775,791 bp assembly. The distribution of chromosome lengths is shown in dark grey with the plot radius scaled to the longest chromosome present in the assembly (62,134,667 bp, shown in red). Orange and pale-orange arcs show the N50 and N90 chromosome lengths (51,702,748 and 19,521,324 bp respectively). The pale grey spiral shows the cumulative chromosome count on a log scale with white scale lines showing successive orders of magnitude. The blue and pale-blue area around the outside of the plot shows the distribution of GC, AT and N percentages in the same bins as the inner plot. A summary of complete, fragmented, duplicated and missing BUSCO genes in the lepidoptera_odb10 set is shown in the top right. An interactive version of this figure is available at https://blobtoolkit.genomehubs.org/view/ilAgrTris1.1/dataset/CAKMRQ01/snail.

The final assembly has a total length of 802 Mb in 62 sequence scaffolds with a scaffold N50 of 51.7 Mb ( Table 1). Most of the assembly sequence (99.83%) was assigned to 23 chromosomal-level scaffolds, representing 22 autosomes (numbered by sequence length) and the Z sex chromosome ( Figure 2– Figure 5; Table 2). Heterozygous inversion was observed on chromosome 1 (19.75–29.97 Mb). A large size differential between haplotypes on several chromosomes was observed, with additional sequence not aligning to comparators. Since difficulty was experienced in reconciling the chromosome 13 longer haplotype with the Hi-C map, 3.3 Mb of the chromosome was left in an alternate assembly.

**Table 1.** Genome data for *A. tristella*, ilAgrTris1.1.

*Project accession data*	
Assembly identifier	ilAgrTris1.1
Species	*Agriphila tristella*
Specimen	ilAgrTris1 (genome assembly, Hi-C)
NCBI taxonomy ID	1594226
BioProject	PRJEB48050
BioSample ID	SAMEA8603174
Isolate information	Male. Thorax (ilAgrTris1, genome assembly); head (ilAgrTris1, Hi-C)
*Raw data accessions*	
PacificBiosciences SEQUEL II	ERR7123973-ERR7123974
10X Genomics Illumina	ERR7113557-ERR7113560
Hi-C Illumina	ERR7113556
*Genome assembly*	
Assembly accession	GCA_928269145.1
*Accession of alternate * *haplotype*	GCA_928269205.1
Span (Mb)	801.8
Number of contigs	149
Contig N50 length (Mb)	15.3
Number of scaffolds	62
Scaffold N50 length (Mb)	51.7
Longest scaffold (Mb)	62.13
BUSCO * genome score	C:98.0%[S:97.4%,D:0.6%],F:0.6%, M:1.4%,n:5,286

*BUSCO scores based on the lepidoptera_odb10 BUSCO set using v5.3.2. C = complete [S single copy, D = duplicated], F = fragmented, M = missing, n = number of orthologues in comparison. A full set of BUSCO scores is available at https://blobtoolkit.genomehubs.org/view/ilAgrTris1.1/dataset/CAKMRQ01/busco.

**Figure 3.**
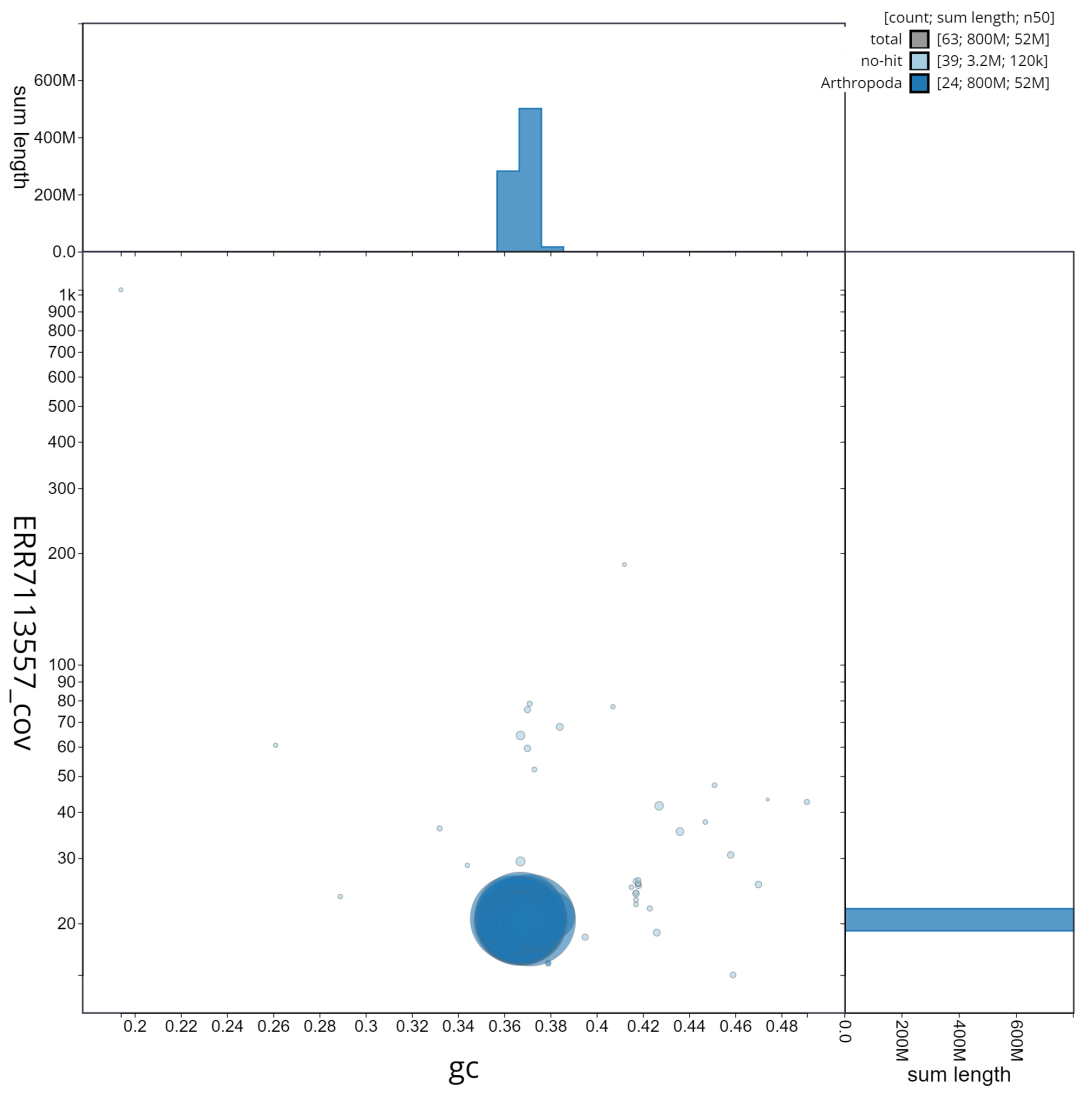
Genome assembly of *A. tristella,* ilAgrTris1.1: GC coverage. BlobToolKit GC-coverage plot. Scaffolds are coloured by phylum. Circles are sized in proportion to scaffold length. Histograms show the distribution of scaffold length sum along each axis. An interactive version of this figure is available at https://blobtoolkit.genomehubs.org/view/ilAgrTris1.1/dataset/CAKMRQ01/blob.

**Figure 4.**
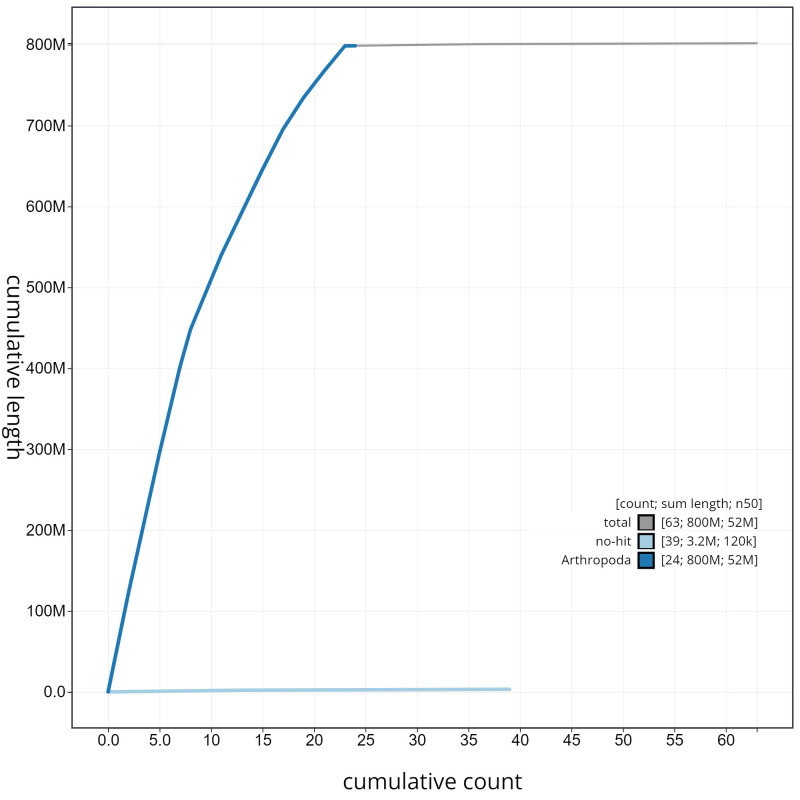
Genome assembly of *A. tristella*, ilAgrTris1.1: cumulative sequence. BlobToolKit cumulative sequence plot. The grey line shows cumulative length for all scaffolds. Coloured lines show cumulative lengths of scaffolds assigned to each phylum using the buscogenes taxrule. An interactive version of this figure is available at https://blobtoolkit.genomehubs.org/view/ilAgrTris1.1/dataset/CAKMRQ01/cumulative.

**Figure 5.**
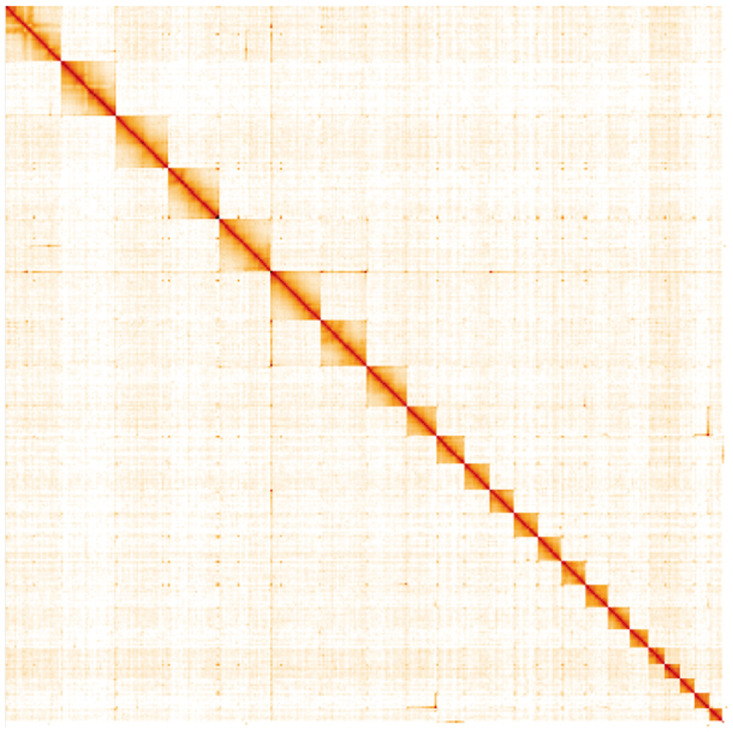
Genome assembly of *A. tristella*, ilAgrTris1.1: Hi-C contact map. Hi-C contact map of the ilAgrTris1.1 assembly, visualised in HiGlass. Chromosomes are arranged in size order from left to right and top to bottom. The interactive Hi-C map can be viewed at https://genome-note-higlass.tol.sanger.ac.uk/l/?d=e5E_CDuZQM6vlEwHYyzUjA.

**Table 2.** Chromosomal pseudomolecules in the genome assembly of *A. tristella*, ilAgrTris1.1.

INSDC accession	Chromosome	Size (Mb)	GC%
OV743429.1	1	62.13	37.1
OV743431.1	2	58.03	36.8
OV743432.1	3	57.33	36.6
OV743433.1	4	56.74	36.7
OV743434.1	5	55.11	36.4
OV743435.1	6	51.7	36.6
OV743436.1	7	45.28	36.6
OV743437.1	8	30.63	37
OV743438.1	9	31.47	36.8
OV743439.1	10	30.18	36.7
OV743440.1	11	27.08	36.3
OV743441.1	12	26.54	36.8
OV743442.1	13	26.08	36.9
OV743443.1	14	26	37.3
OV743444.1	15	25.48	36.4
OV743445.1	16	24.34	37.2
OV743446.1	17	19.6	36.5
OV743447.1	18	19.52	37.4
OV743448.1	19	16.52	38
OV743449.1	20	16.29	36.7
OV743450.1	21	15.62	36.9
OV743451.1	22	15.29	37.1
OV743430.1	Z	61.58	36.7
OV743452.1	MT	0.02	19.6
-	Unplaced	3.21	40.4

The assembly has a BUSCO v5.3.2 ( Manni *et al.*, 2021) completeness of 98.0% (single 97.4%, duplicated 0.6%) using the lepidoptera_odb10 reference set ( *n* = 5,286). While not fully phased, the assembly deposited is of one haplotype. Contigs corresponding to the second haplotype have also been deposited.

## Methods

### Sample acquisition and nucleic acid extraction

A single male *A. tristella* specimen (ilAgrTris1) was collected in Wytham Woods, Oxford, Berkshire, UK (latitude 51.772, longitude –1.338) by Douglas Boyes (University of Oxford), using a light trap. The specimen was identified by Douglas Boyes and snap-frozen on dry ice.

DNA was extracted at the Tree of Life laboratory, Wellcome Sanger Institute. The ilAgrTris1 sample was weighed and dissected on dry ice with tissue set aside for Hi-C sequencing. Thorax tissue was disrupted using a Nippi Powermasher fitted with a BioMasher pestle. Fragment size analysis of 0.01–0.5 ng of DNA was then performed using an Agilent FemtoPulse. High molecular weight (HMW) DNA was extracted using the Qiagen MagAttract HMW DNA extraction kit. Low-molecular weight DNA was removed from a 200 ng aliquot of extracted DNA using 0.8X AMpure XP purification kit prior to 10X Chromium sequencing; a minimum of 50 ng DNA was submitted for 10X sequencing. HMW DNA was sheared into an average fragment size of 12–20 kb in a Megaruptor 3 system with speed setting 30. Sheared DNA was purified by solid-phase reversible immobilisation using AMPure PB beads with a 1.8X ratio of beads to sample to remove the shorter fragments and concentrate the DNA. The concentration of the sheared and purified DNA was assessed using a Nanodrop spectrophotometer and Qubit Fluorometer and Qubit dsDNA High Sensitivity Assay kit. Fragment size distribution was evaluated by running the sample on the FemtoPulse system.

### Sequencing

Pacific Biosciences HiFi circular consensus and 10X Genomics Chromium read cloud sequencing libraries were constructed according to the manufacturers’ instructions. Sequencing was performed by the Scientific Operations core at the Wellcome Sanger Institute on Pacific Biosciences SEQUEL II (HiFi) and Illumina NovaSeq 6000 (10X) instruments. Hi-C data were generated in the Tree of Life laboratory from head tissue of ilAgrTris1 using the Arima v2 kit and sequenced on a NovaSeq 6000 instrument.

### Genome assembly

Assembly was carried out with Hifiasm ( Cheng *et al.*, 2021); haplotypic duplication was identified and removed with purge_dups ( Guan *et al.*, 2020). One round of polishing was performed by aligning 10X Genomics read data to the assembly with longranger align, calling variants with freebayes ( Garrison & Marth, 2012). The assembly was then scaffolded with Hi-C data ( Rao *et al.*, 2014), using YaHS ( Zhou *et al.*, 2022). The assembly was checked for contamination as described previously ( Howe *et al.*, 2021). Manual curation was performed using HiGlass ( Kerpedjiev *et al.*, 2018) and PretextView ( Harry, 2022). The mitochondrial genome was assembled using MitoHiFi ( Uliano-Silva *et al.*, 2021), which performs annotation using MitoFinder ( Allio *et al.*, 2020). The genome was analysed and BUSCO scores were generated within the BlobToolKit environment ( Challis *et al.*, 2020). Table 3 contains a list of all software tool versions used, where appropriate.

**Table 3.** Software tools used.

Software tool	Version	Source
Hifiasm	0.15.3	( Cheng *et al.*, 2021)
purge_dups	1.2.3	( Guan *et al.*, 2020)
YaHS	1.0	( Zhou *et al.*, 2022)
longranger align	2.2.2	https://support.10xgenomics. com/genome-exome/software/ pipelines/latest/advanced/other- pipelines
freebayes	1.3.1-17- gaa2ace8	( Garrison & Marth, 2012)
MitoHiFi	2.0	( Uliano-Silva *et al.*, 2021)
HiGlass	1.11.6	( Kerpedjiev *et al.*, 2018)
PretextView	0.2.x	https://github.com/wtsi-hpag/ PretextView
BlobToolKit	3.2.6	( Challis *et al.*, 2020)

### Ethics/compliance issues

The materials that have contributed to this genome note have been supplied by a Darwin Tree of Life Partner. The submission of materials by a Darwin Tree of Life Partner is subject to the Darwin Tree of Life Project Sampling Code of Practice. By agreeing with and signing up to the Sampling Code of Practice, the Darwin Tree of Life Partner agrees they will meet the legal and ethical requirements and standards set out within this document in respect of all samples acquired for, and supplied to, the Darwin Tree of Life Project. Each transfer of samples is further undertaken according to a Research Collaboration Agreement or Material Transfer Agreement entered into by the Darwin Tree of Life Partner, Genome Research Limited (operating as the Wellcome Sanger Institute), and in some circumstances other Darwin Tree of Life collaborators.

## Data Availability

European Nucleotide Archive: *Agriphila tristella* (common grass-veneer). Accession number PRJEB48050; https://identifiers.org/ena.embl/PRJEB48050 ( Wellcome Sanger Institute, 2022) The genome sequence is released openly for reuse. The *A. tristella* genome sequencing initiative is part of the Darwin Tree of Life (DToL) project. All raw sequence data and the assembly have been deposited in INSDC databases. The genome will be annotated and presented through the Ensembl pipeline at the European Bioinformatics Institute. Raw data and assembly accession identifiers are reported in Table 1.

## Author information

Members of the University of Oxford and Wytham Woods Genome Acquisition Lab are listed here: https://doi.org/10.5281/zenodo.4789928.

Members of the Darwin Tree of Life Barcoding collective are listed here: https://doi.org/10.5281/zenodo.4893703.

Members of the Wellcome Sanger Institute Tree of Life programme are listed here: https://doi.org/10.5281/zenodo.4783585.

Members of Wellcome Sanger Institute Scientific Operations: DNA Pipelines collective are listed here: https://doi.org/10.5281/zenodo.4790455.

Members of the Tree of Life Core Informatics collective are listed here: https://doi.org/10.5281/zenodo.5013541.

Members of the Darwin Tree of Life Consortium are listed here: https://doi.org/10.5281/zenodo.4783558.
