# Supplementary material for: The genome sequence of the common grass-veneer, Agriphila tristella (Denis & Schiffermüller, 1775)
Source: Wellcome Open Res. 2022 Dec 16;7:304. [Version 1] doi: 10.12688/wellcomeopenres.18568.1 (PMC11128054; doi:10.12688/wellcomeopenres.18568.1)
Supplement: Peer review history

# Reviewer report: Reviewer response for version 1

**DOI:** 10.21956/wellcomeopenres.20589.r57003

**Published:** 2024-06-12

**Authors:** Wei Zhang

**Affiliations:** 1 State Key Laboratory of Protein and Plant Gene Research, Peking-Tsinghua Center for Life Sciences, Academy for Advanced Interdisciplinary Studies, School of Life Sciences, Peking University, Beijing, China

This manuscript reports a genome assembly at the chromosome level of the common grass-veneer *Agriphila tristella* with the Z chromosome and the mitochondrial genome assembled. This genome assembly has an N50 value of 51.7 Mb and a total length of 802 Mb. It also has considerable BUSCO statistics and annotations. The methods section is well written. Besides, this manuscript also describes the morphology, life history, and distribution area of the focal species, which contributes to the better use of this genome assembly. I think the overall quality of this genome assembly is valid and will benefit relevant studies.

Are sufficient details of methods and materials provided to allow replication by others?

Yes

Is the rationale for creating the dataset(s) clearly described?

Yes

Are the datasets clearly presented in a useable and accessible format?

Yes

Are the protocols appropriate and is the work technically sound?

Yes

Reviewer Expertise:

evolutionary genomics

I confirm that I have read this submission and believe that I have an appropriate level of expertise to confirm that it is of an acceptable scientific standard.

# Reviewer report: Reviewer response for version 1

**DOI:** 10.21956/wellcomeopenres.20589.r70181

**Published:** 2024-05-25

**Authors:** Annabel Whibley

**Affiliations:** 1 The University of Auckland, Auckland, Auckland, New Zealand; 2 Bragato Research Institute, Blenheim, New Zealand

Review of the genome assembly of *Agriphila tristella *by Douglas Boyes and colleagues at the University of Oxford and Wellcome Trust Sanger Centre. The authors have assembled another extremely high-quality genome and provided comprehensive and clearly structured information to support this effort. Yet again, the well-informed natural history detail that is included provides very helpful framing. Methods, analyses and reporting are all exemplary.

 I note two omissions in this report compared to an earlier report that I reviewed: (1) Genome annotation- is there a reason why this assembly hasn’t been run through the Ensembl rapid annotation pathway? (2) A link to the metadata, such as kmer spectra- which can be a really useful resource.

 The level of heterozygosity in the genome individual seems quite marked and, from the haplotype size differences recorded, it would appear that structural variation may be an important feature of genome biology in this species.  Whilst to explore this in detail is probably beyond the scope of these reports, it would have been nice to have these “quirks” also presented as tables/figures. As the reports of the primary assemblies become increasingly standardized (and these short reports are models in the field), as a collective I would love to see the DToL and its partners explore ways to effectively disseminate learnings from unexpected and challenging findings.

Are sufficient details of methods and materials provided to allow replication by others?

Yes

Is the rationale for creating the dataset(s) clearly described?

Yes

Are the datasets clearly presented in a useable and accessible format?

Yes

Are the protocols appropriate and is the work technically sound?

Yes

Reviewer Expertise:

Genomics

I confirm that I have read this submission and believe that I have an appropriate level of expertise to confirm that it is of an acceptable scientific standard.

# Reviewer report: Reviewer response for version 1

**DOI:** 10.21956/wellcomeopenres.20589.r70179

**Published:** 2023-11-29

**Authors:** Kuppusamy Sivasankaran

**Affiliations:** 1 Loyola College, Chennai, India

I would like to appreciate the authors for assembling the *Agriphila tristella *(Denis & Schiffermuller, 1775) of whole genome sequence. The authors sequenced almost 800 million base pairs in the genome assembly using 51 scaffolds. The authors have properly submitted the sequence in the public database. This robust data will be useful for the phylogenomic researchers.   

 Suggestions:

 In the abstract the first sentence can be rewritten as “The genome of *Agriphila tristella* (Insecta: Lepidoptera: Crambidae) was sequenced and assembled”.  

 The last sentence of the abstract can be changed as “The mitochondrial genome was also annotated and is 15.3 kilobases in size”.

 Under the subheading “Background” Third paragraph of the first line can be modified as “The genome of *A. tristella *was sequenced…………………”

 Under the subheading “Genome sequence report” first paragraph sixth line” with chromosome conformation” It can be changed as “with chromosome confirmation”

 Query:

 Why the authors haven’t included the details about the annotation of Protein-coding genes, non-coding sequences, number of gene transcripts in the tables.

 The manuscript is well prepared, and it can be accepted for indexing.

Are sufficient details of methods and materials provided to allow replication by others?

Yes

Is the rationale for creating the dataset(s) clearly described?

Yes

Are the datasets clearly presented in a useable and accessible format?

Yes

Are the protocols appropriate and is the work technically sound?

Yes

Reviewer Expertise:

Phylogenetic analysis of lepidopteran moths using complete mitochondrial genome sequence

I confirm that I have read this submission and believe that I have an appropriate level of expertise to confirm that it is of an acceptable scientific standard.
